# Association of *ERCC1* Gene Polymorphisms (rs3212986 and rs11615) With the Risk of Lung Cancer in a Population From Southeast Iran

**DOI:** 10.34172/jrhs.2024.166

**Published:** 2024-09-30

**Authors:** Ali Khalouei, Yaser Masoumi-Ardakani, Abdollah Jafarzaheh, Behjat Kalantari Khandani, Farnaz Sedghy, Arezu Khosravi Mashizi, Mohammad Mehdi Yaghoobi, Mohammadreza Zangouey, Beydolah Shahouzehi

**Affiliations:** ^1^Physiology Research Center, Institute of Neuropharmacology, Kerman University of Medical Sciences, Kerman, Iran; ^2^Student Research Committee, Kerman University of Medical Sciences, Kerman, Iran; ^3^Department of Immunology, Medical School, Kerman University of Medical Sciences, Kerman, Iran; ^4^Department of Hematology and Oncology, Faculty of Medicine, Kerman University of Medical Sciences, Kerman, Iran; ^5^Department of Immunology, Afzalipour Faculty of Medicine, Kerman University of Medical Sciences, Kerman, Iran; ^6^Research Department of Biotechnology, Institute of Science and High Technology and Environmental Sciences, Graduate University of Advanced Technology, Kerman, Iran; ^7^Cardiovascular Research Center, Basic and Clinical Physiology Sciences, Kerman University of Medical Sciences, Kerman, Iran

**Keywords:** Lung cancer, Chemotherapy, ERCC1 gene, Polymorphism, PCR-RFLP, Pharmacogenetic studies

## Abstract

**Background:** Polymorphisms within the excision repair cross-complementation group 1 (*ERCC1*), an essential component of DNA repair mechanisms, have been associated with various malignancies. This study aimed to evaluate the association of the single-nucleotide polymorphisms (SNPs) rs3212986 and rs11615 within the *ERCC1* gene in non-small cell lung cancer (NSCLC) patients.

**Study Design:** A case-control study.

**Methods:** Genomic DNA was extracted from the peripheral blood samples of 83 NSCLC patients and 119 healthy individuals. The genetic diversity of SNPs rs3212986 and rs11615 was determined using the polymerase chain reaction-restriction fragment length polymorphism (PCR-RFLP) method. The RFLP results were confirmed through sequencing.

**Results:** The TT genotype of the rs11615 SNP was associated with a higher risk of NSCLC development (odds ratio: 3.900, 95% confidence interval: 0.603, 22.866, P=0.050). Furthermore, the AA genotype of rs3212986 was related to a higher risk of NSCLC development (OR: 2.531, 95% CI: 1.017, 6.300, *P*=0.046). A significant association was observed between smoking and lung cancer (OR: 3.072, 95% CI: 1.715, 5.503, *P*<0.001). Moreover, among non-smokers, there was an association between lung cancer risk and the AA (OR: 6.825, 95% CI: 1.722, 27.044, *P*=0.006) and AC (OR: 2.503, 95% CI: 0.977, 6.412, *P*=0.056) genotypes of rs3212986. However, no correlation was found between the genotypes of these SNPs and patients’ sensitivity to cisplatin and carboplatin (*P* ˃ 0.05).

**Conclusion:** The rs11615-related TT genotype and the rs3212986-related AA genotype may be associated with a higher risk of lung cancer development.

## Background

 Lung cancer remains one of the most prevalent cancers and the leading cause of cancer-related death worldwide.^1^ The incidence of lung cancer is increasing, especially in developing countries. In 2018, approximately 2.09 million new cases of lung cancer were reported, resulting in around 1.76 million deaths.^2^ Lung cancer is primarily classified into non-small cell lung cancer (NSCLC) and small cell lung types, accounting for approximately 85.0% and 15.0% of all malignant lung cancer cases, respectively.^3^ NSCLC cases are diagnosed in approximately two-thirds of patients’ primary at advanced stages (stage III or IV), with a 5-year survival rate of less than 15% due to lower response rates to standard treatment options.^4^ Common treatments for lung cancer include surgery, radiation, chemotherapy, and immunotherapy. However, considering that NSCLC is frequently diagnosed late, platinum-based chemotherapy or chemoradiotherapy is often the only option for most patients.^5^ Inter-individual diversity in response to medication was observed in NSCLC patients with the same disease stage and treatment regimen, indicating that genetic factors may significantly influence treatment efficacy.^6^

 Chemotherapy using platinum compounds induces DNA damage by generating DNA-platinum adducts that activate the cell’s DNA repair machinery.^7,8^ The main mechanisms to protect DNA from mutagens and repair damage caused by various endogenous and exogenous agents include nucleotide excision repair, double-strand break repair, and base excision repair. Importantly, the excision repair cross-complementation group 1 (*ERCC1*) gene encodes a specific endonuclease within the nucleotide excision repair system that catalyzes the DNA strand breaks at the 5’ end of the damaged site. DAN double-strand breaks and cross-linking damages are also repaired by the *ERCC1*-encoded endonuclease.^4,9,10^

 Genetic mutations within DNA repair genes can alter the expression, structure, and function of associated proteins, serving as important prognostic indicators for malignancies.^1^ Several studies suggest that the presence of certain functional polymorphisms within the *ERCC1* gene may decrease DNA repair capabilities, thereby increasing the risk of various malignancies.^11-14^ Several other studies have also pointed to an association between *ERCC1* variants and clinical outcomes in NSCLC patients.^15,16^ Regarding the carcinogenic potential of *ERCC1* polymorphisms, these changes might serve as useful prognostic biomarkers in lung cancer. To the best of our knowledge, there is no report on the associations between polymorphisms of the *ERCC1* gene and lung cancer in Iranian populations. Therefore, the current study has focused on the association of single-nucleotide polymorphisms (SNPs) rs3212986 and rs11615 within the *ERCC1* gene with NSCLC in a southeastern Iranian population.

## Methods

###  Study subjects 

 In this case-control study, the sample size was determined by G*Power software, version 3.1.9.2. Considering the alpha error of 5%, test power of 80%, effect size of 0.218, and the degree of freedom of 2, a sample size of 204 was selected for this research.^17,18^ This study involved 83 NSCLC patients and 119 healthy subjects. NSCLC patients were recruited among those referred to the Javad Al-Aeme Clinic of Kerman University of Medical Sciences from May 2020 to September 2021. NSCLC was diagnosed by a respiratory specialist based on lung examination. Patient inclusion criteria were histological or cytological confirmed diagnosis of NSCLC. The healthy individuals were selected from blood donors at a regional blood transfusion center in Kerman, and none had a history of respiratory disease or other related disorders. Based on the data in Table 1, there was no significant difference between the demographic features of the study groups. Approval for the study protocol was obtained from the Ethics Committee of Kerman University of Medical Sciences (code IR.KMU.REC.1399.072). Furthermore, all participants were recruited after signing a written informed consent form. Peripheral blood samples (5 mL) were collected from each participant to determine the polymorphisms.

**Table 1:** Demographic and clinicopathological characteristics for malignant patients

**Characteristics**	**Control (n=119)**		**Case (n=83)**		***P*** ** value**
	**Number**	**Percent**	**Number**	**Percent**	
Gender					
Men	83	69.74	64	77.10	0.354
Women	36	30.25	19	22.89	
Smoking status					0.001
Smokers	38	31.93	49	59.03	
Non-smokers	81	68.06	34	40.96	
Tumor stage					
I	-		0	0	
II	-		0	0	
III	-		26	31.32	
IV	-		57	68.67	
Histology					
Adenocarcinoma	-		52	62.65	
Squamous cell carcinoma	-		29	31.32	
Large cell carcinoma	-		2	2.40	
Response to chemotherapy					
Complete response + partial response	-		16	40	
Stable disease + progressive disease	-		24	60	

###  Chemotherapy regimen

 All patients were treated with cisplatin- or carboplatin-based chemotherapy every 3 weeks. After a median follow-up of 15 months, responses to treatment were determined and defined as NSCLC patients with complete response (CR) and partial response (PR) to chemotherapy. No-responders were defined as stable disease (SD) and progressive disease (PD).

###  Genomic DNA extraction 

 Genomic DNA was isolated from peripheral blood using a salting-out technique. Spectrophotometry (Eppendorf, Germany) was used to determine DNA quantities and purity according to the measurements of the absorbance at 260 nm and 280 nm. Extracted DNA samples were stored at -20°C until use.

###  Genotyping assay

 The SNPs rs11615 and rs3212986 in *ERCC1* were determined by the polymerase chain reaction-restriction fragment length polymorphism (PCR-RFLP) method. The PCR was performed as 1 μL of prepared DNA, 3 μL of the PCR buffer (10x), 0.3 μL of the Taq DNA polymerase (5 U/mM), 1.5 μL of MgCl2 (stock concentration 1.5 mM), 0.5 μL dNTPs (stock concentration of 10 mM), 2 μL of each primer (300 nmol), and distilled water up to 30 μL. The forward (5′-GGTGCAAGAAGAGGTGGAG-3′ and 5′-AAGAAGCAGAGTCAGGAAAGC-3′) and reverse (5′-TCAGATCCCCAGGAGTCC-3′ and 5′-ACCCCACTCTAGATTTACCCAG-3) primers were used to amplify the regions around the SNPs rs11615 and rs3212986, respectively. The amplification program was designed as an initial denaturation step (95 ˚C for 5 minutes), followed by 40 cycles of denaturation (94 °C for 15 seconds), annealing (60 °C for 25 seconds), and extension (72°C for 30 seconds). Additionally, a final extension step was performed (72 °C for 10 minutes) to complete the PCR.

**Figure 1:**
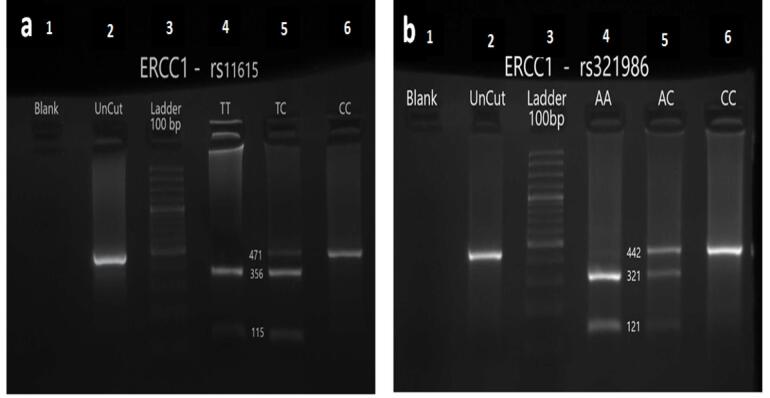
Agarose gel electrophoresis of amplified PCR product of *ERCC1* rs11615 (a) and *ERCC1* rs321986 (b). Columns: (1) Blank, (2) Uncut PCR product, (3) Ladder, (4) TT (a) and AA (b) Genotype, (5) TC (a) and AC (b) Genotype, and (6) CC genotype. *Note*. PCR: Polymerase chain reaction.

**Figure 2:**
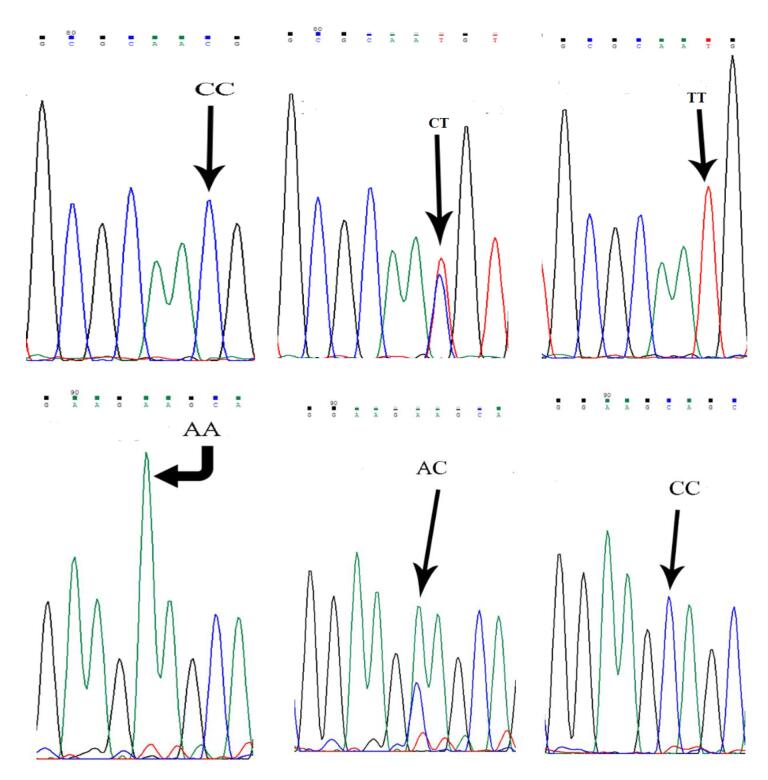
Partial DNA sequence results regarding SNPs rs11615 (a) and rs3212986 (b). *Note*. SNP: single-nucleotide polymorphism

 The amplified PCR products contain SNPs rs11615 and rs3212986 with lengths of 471 bp and 442 bp, respectively. Subsequently, PCR products (5 μL) related to SNPs rs11615 and rs3212986 were digested overnight (37 °C) in the presence of 0.75 unit/reaction of BsrDI and MBOII restriction endonuclease, respectively. Considering that the BsrDI enzyme has exclusively one restriction site in the SNP rs11615-related PCR product, thus the CC genotype has two DNA fragments of 184 bp and 319 bp, whereas the AA genotype has a single undigested DNA fragment (503 bp), and the heterozygous CA genotype has three DNA fragments (503 bp, 319 bp, and 184 bp). The MBOII enzyme has only one restriction site in the PCR product associated with the rs3212986 SNP; thus, the CC genotype has two DNA fragments of 184 bp and 319 bp. However, the AA genotype has a single undigested DNA fragment (503 bp), and the heterozygous CA genotype has three DNA fragments (503 bp, 319 bp, and 184 bp). After adding 2 μL of DNA safe stain (Kowsar Biotech Company, Iran) to enzyme-treated products, they were electrophoresed on a 3% agarose gel and evaluated with Chemi-Doc model XRS (Bio-Rad, USA; Figure 1). Finally, the sequencing was performed on 22 samples for each SNP to confirm the RFLP results. Partial DNA sequence results for SNPs rs11615 and rs3212986 are shown in Figure 2.

###  Statistical analysis

 The data were analyzed by statistical SPSS software (version 26, Chicago, IL, USA). Differences in variables were analyzed by using appropriate statistical tests, including logistic regression, independent sample* t*-test, and the chi-square (*χ*^2^). *P* values less than 0.05 were considered statistically significant.

## Results

###  Patient characteristics 

 The clinicopathological characteristics of the malignant patients are provided in Table 1. The study was performed on 83 advanced NSCLC patients, including 64 men and 19 women, with a mean ( ± standard deviation) age of 56.26 ( ± 1.48) years. Of the patients, 26 were at stage III, and 57 were at stage IV. Forty-nine of all NSCLC patients were smokers. Of the 40 patients evaluated for the treatment response, 24 (60%) showed SD + PD (non-responsive state) to chemotherapy, of whom 10 (25%) died (PD). Among 16 (40%) responders (CR + PR), 3 (7.5%) had a CR.

 The control group included 119 normal or healthy people (83 men and 36 women, with a mean [ ± SD] age of 54.87 [ ± 1.18] years). The age was not significantly different between case and control groups (*P* = 0.463).

###  Genetic variation of ERCC1 rs11615 single-nucleotide polymorphism

 The genotypes and allele frequencies of the *ERCC1*-related rs11615 SNP in malignant and control groups are presented in Table 2. The frequencies of CC, TC, and TT genotypes at the rs11615 of the *ERCC1* gene were 36.14%, 53.01%, and 10.840% in lung cancer patients, as well as 32.77%, 64.70%, and 2.52% in the control group. A significant difference was found between lung cancer patients and controls in the frequency of the CC, TC, and TT genotypes at the rs11615 SNP (*P* = 0.031). The difference in the frequency of T and C alleles between the malignant patients and controls was not significant. Considering the CC genotype as a criterion, it was found that the TT genotype was associated with a higher risk of lung cancer development (odds ratio: 3.9, 95% confidence interval: 0.603, 22.866; *P* = 0.055, Table 2).

**Table 2:** Frequency of genotypes and alleles at SNP rs11615 in the *ERCC1* gene in malignant patients and healthy control group

**SNP rs11615**	**Lung Cancer**		**Healthy Group**		***P*** ** value**	**OR (95% CI)**	***P*** ** value**
	**Number**	**Percent**	**Number**	**Percent**			
Genotypes					0.031		
CC	30	36.14	39	32.77		Ref.	
TC	44	53.02	77	64.70		0.742 (0.406, 1.358)	0.334
TT	9	10.84	3	2.52		3.900 (0.603, 22.866)	0.055
Alleles					0.750		
C	104	62.65	155	65.12		Ref.	
T	62	37.35	83	34.87		1.113 (0.737, 1.681)	0.610

*Note*. SNP: single-nucleotide polymorphism; CI: Confidence interval; OR: Odds ratio.

###  Genetic variation of ERCC1 rs3212986 single-nucleotide polymorphism

 The genotypes and allele frequencies of the *ERCC1*-related rs3212986 SNP in malignant and control groups are listed in Table 3. The frequencies of CC, AC, and CC genotypes at the rs3212986 of the *ERCC1* gene were 38.55%, 43.37%, and 60.24% in lung cancer patients, as well as 45.37%, 46.21%, and 8.40% in the control group. Differences in the frequencies of CC, AC, and CC genotypes between the malignant and control groups were not significant. Similarly, there was no significant difference in allele A and allele C frequencies between the malignant and control groups. Considering the CC genotype as a reference, it was revealed that the AA genotype was associated with a higher risk of lung cancer development (OR: 2.531, 95% CI: 1.017, 6.300; *P*= 0.046, Table 3).

**Table 3:** Frequency of genotypes and alleles at SNP rs3212986 in the *ERCC1* gene in malignant patients and healthy control group

**SNP rs3212986**	**Lung Cancer**		**Healthy Group**		***P*** ** value**	**OR (95% CI)**	***P*** ** value**
	**Number**	**Percent**	**Number**	**Percent**			
Genotypes					0.116		
CC	32	38.56	54	45.37		Ref.	
AC	36	43.37	55	46.22		1.105 (0.602, 2.026)	0.748
AA	15	18.07	10	8.41		2.531 (1.017, 6.300)	0.046
Alleles					0.087		
C	100	60.25	163	68.48		Ref.	
A	66	39.75	75	31.52		0.697 (0.461, 1.055)	0.088

*Note*. SNP: single-nucleotide polymorphism; CI: Confidence interval; OR: Odds ratio.

###  Genetic variation of ERCC1 single-nucleotide polymorphisms and smoking

 A significant association was observed between smoking and lung cancer (OR: 3.072, 95% CI: 1.715, 5.503; *P* < 0.001). The relationship between genotype and the risk of lung cancer was further analyzed in the malignant and healthy control groups based on smoking status, and there were no significant differences in this regard (Table 4). Based on the results, there was an association between the risk of lung cancer and AA (OR: 6.825, 95% CI: 1.722, 27.044; *P*= 0.006) and AC (OR: 2.503, 95% CI: 0.977, 6.412; *P*= 0.056) genotypes of the non-smoking group (Table 4).

**Table 4:** Frequency of Genotypes at SNPs rs11615 and rs3212986 in the *ERCC1* Gene in Malignant Patients and Healthy Control Group Based on Smoking Status

**Genotypes**	**Smokers**				**Non-smokers**			
	**Case**	**Control**	**OR (95% CI)**	***P *** **value**	**Case**	**Control**	**OR (95% CI)**	***P *** **value**
rs11615								
TT	5	1	4.062 (0.420, 39.257)	0.226	4	2	3.714 (0.603, 22.866)	0.157
TC	28	24	0.948 (0.381, 2.361)	0.909	16	53	0.561 (0.238, 1.321)	0.186
CC	16	13	Ref.		14	26	Ref.	
rs3212986								
AA	8	5	1.001 (0.275, 3.634)	0.999	7	5	6.825 (1.722, 27.044)	0.006
AC	17	18	0.590 (0.234, 1.489)	0.264	19	37	2.503 (0.977, 6.412)	0.056
CC	24	15	Ref.		8	39	Ref.	

*Note*. SNP: single-nucleotide polymorphism; CI: Confidence interval; OR: Odds ratio.

###  Genetic variation of ERCC1 single-nucleotide polymorphisms and sensitivity to cisplatin and carboplatin

 The frequencies of the genotypes of *ERCC1*-related SNPs (rs11615 and rs3212986) based on sensitivity to cisplatin and carboplatin are presented in Table 5. No correlation with the genotypes was found in these SNPs and sensitivity to cisplatin and carboplatin (*P* = 0.520 and *P* = 0.458, respectively) in malignant patients.

**Table 5:** Frequency of genotypes at SNPs rs11615 and rs3212986 in the *ERCC1* gene in malignant patients based on sensitivity to cisplatin and carboplatin

	**Total Frequency**	**Responders**	**Non-responders**	**OR (95% CI)**	***P*** ** value**
		**PR+CR**	**SD+PD**		
Genotype (rs11615)					
Cisplatin					
TT	2	1	1	1.001 (0.041, 24.547)	0.999
TC	19	6	13	2.166 (0.334, 14.057)	0.418
CC	6	3	3	Ref.	
Carboplatin					
TT	3	2	1	0.375 (0.022, 6.348)	0.497
TC	3	1	2	1.500 (0.089, 25.392)	0.779
CC	7	3	4	Ref.	
Genotype (rs3212986)					
Cisplatin					
AA	5	3	2	0.667 (0.069, 6.409)	0.725
AC	14	3	11	3.667 (0.5557, 24.132)	0.177
CC	8	4	4	Ref.	
Carboplatin					
AA	4	1	3	3.000 (0.188, 47.963)	0.437
AC	3	2	1	0.500 (0.028, 8.952)	0.638
CC	6	3	3	Ref.	

*Note*. SNP: single-nucleotide polymorphism; CI: Confidence interval; OR: Odds ratio. CR: Complete response; PR: Partial response; SD: Stable disease; PD: Progressive disease.

## Discussion

*ERCC1* plays a fundamental role in DNA repair, and over 100 polymorphisms have been identified in the *ERCC1* gene that can influence the structure and function of the encoded protein.^19^ In this case-control study, the rs11615-related TT genotype and the s3212986-related AA genotype of the *ERCC1* gene were associated with a higher risk of lung cancer development in Iranian patients, with advanced NSCLC cases who have been treated with platinum-based chemotherapy. Furthermore, the relationship between the rs11615 SNP genotype and lung cancer risk was analyzed based on smoking status, revealing an association between lung cancer risk and AA and AC genotypes in non-smokers. Previous studies reported a correlation between *ERCC1* polymorphisms and NSCLC susceptibility, which supports our results. For instance, Yu et al showed that NSCLC patients with the rs3212986 AA genotype in the *ERCC1* gene have an increased risk of lung cancer, particularly among smokers.^20^ Gao also demonstrated that *ERCC1* genotype rs11615 TT and genotype rs3212986 AA contribute to increased mortality risk from NSCLC. Additionally, a study involving Chinese patients indicated that the presence of the AA genotype at *ERCC1 *rs3213986 was associated with an increased risk of lung cancer, particularly in smokers.^21^ Other studies have suggested the association between various *ERCC1* genotypes and the risk of lung cancer. Chaszczewska-Markowska et al confirmed the role of the rs11615 T allele and the rs3212986 GG genotype in NSCLC susceptibility.^1^ Likewise, two additional studies highlighted the predictive significance of the *ERCC1* rs3212986 polymorphism in NSCLC patients.^19,22^

 There is also evidence that certain SNPs within the *ERCC1* gene can lead to decreased levels of *ERCC1* mRNA and protein, which potentially impair cellular repair ability and ultimately promote cancer development.^23-26^ Moreover, several studies have confirmed a relationship between *ERCC1* gene polymorphisms and NSCLC treatment efficacy.^10,11,15,27,28^ The results of a studyin Chinese patients revealed that individuals with the rs11615 TT genotype and the T allele or the rs3212986 AA genotype and the A allele had significantly lower response rates to treatment. As a result, these SNPs could serve as predictors of treatment response and may be associated with increased mortality in patients with advanced NSCLC.^21^ It has been suggested that rs11615 (*ERCC1*) and rs3738948 (*ERCC3*) can influence the efficacy of platinum-based chemotherapy in NSCLC patients.^29^ On the other hand, some studies have failed to find a significant relationship between *ERCC1* gene polymorphisms and treatment efficacy in NSCLC patients receiving cisplatin-based chemotherapy. The results of a study among Iranian patients with colorectal and gastric cancers showed no significant association between genetic variations in the *ERCC1* rs11615 SNP and response to oxaliplatin-based chemotherapy.^30^ Consistent with these findings, no significant effect of *ERCC1* polymorphisms on treatment response was observed in NSCLC patients receiving cisplatin-based chemotherapy. These contradictory results may be due to differences in population, case selection, and study design. In our study, the relatively small number of NSCLC patients with homozygous genotypes may affect the power of statistical analysis to determine an association between *ERCC1* polymorphisms and treatment response. Several limitations of the current study require consideration. First, the relatively small sample size, especially of lung cancer cases, restricted the statistical power for subgroup analysis; thus, the results should be confirmed with studies of larger populations. Second, this study only evaluated the risk of lung cancer, but investigating prognostic potential, especially by measuring survival parameters, could be valuable. Finally, further experiments are needed to elucidate the specific contribution of *ERCC1* polymorphisms to the susceptibility to lung cancer susceptibility as well as to reveal other functional SNPs involved in DNA repair pathways.

**Highlights**

- ERCC1 rs11615 and rs3212986 polymorphisms are associated with a higher risk of lung cancer.
- SNPs rs11615 and rs3212986 may serve as useful predictive markers to identify individuals at increased risk of lung cancer.
- The TT genotype of the rs11615 SNP was associated with a higher risk of NSCLC development.

## Conclusion

 In summary, the findings revealed that the *ERCC1 *rs11615 and rs3212986 polymorphisms are associated with a higher risk of lung cancer. These SNPs may serve as useful predictive markers to identify individuals at increased risk of lung cancer.

## Authors’ Contribution

**Conceptualization:** Yaser Masoumi-Ardakani, Abdollah Jafarzaheh, Behjat Kalantari Khandani.

**Data curation:** Ali Khalouei, Arezu Khosravi Mashizi, Mohammadreza Zangouey, Mohammad Mehdi Yaghoobi, Beydolah Shahouzehi.

**Formal analysis:** Mohammadreza Zangouey, Ali Khalouei.

**Funding acquisition:** Yaser Masoumi-Ardakani.

**Investigation:** Ali Khalouei, Yaser Masoumi-Ardakani.

**Methodology:** Ali Khalouei, Arezu Khosravi Mashizi, Mohammad Mehdi Yaghoobi, Beydolah Shahouzehi.

**Project administration:** Yaser Masoumi-Ardakani.

**Resources: **Farnaz Sedghy, Behjat Kalantari Khandani.

**Software:** Ali Khalouei, Mohammadreza Zangouey.

**Supervision:** Yaser Masoumi-Ardakani.

**Validation:** Yaser Masoumi-Ardakani, Farnaz Sedghy, Arezu Khosravi Mashizi, Mohammad Mehdi Yaghoobi.

**Visualization:** Ali Khalouei, Arezu Khosravi Mashizi.

**Writing–original draft**: Yaser Masoumi-Ardakani, Abdollah Jafarzaheh, Behjat Kalantari Khandani, Farnaz Sedghy, Arezu Khosravi Mashizi, Mohammad Mehdi Yaghoobi,Mohammadreza Zangouey, Beydolah Shahouzehi.

**Writing–review & editing:** Yaser Masoumi-Ardakani, Farnaz Sedghy.

## Competing Interests

 The authors declare that there is no conflict of interests.

## Ethical Approval

 The current study was approved by the Ethics Committee of Kerman University of Medical Sciences and Health Services (IR.KMU.REC.1399.072), and all procedures were in accordance with standards set by the Declaration of Helsinki (1964).

## Funding

 This study was financially supported by the Kerman Student Research Committee (Grant number: 98000685).
